# Computational fluid dynamics (CFD) using porous media modeling predicts recurrence after coiling of cerebral aneurysms

**DOI:** 10.1371/journal.pone.0190222

**Published:** 2017-12-28

**Authors:** Yasuyuki Umeda, Fujimaro Ishida, Masanori Tsuji, Kazuhiro Furukawa, Masato Shiba, Ryuta Yasuda, Naoki Toma, Hiroshi Sakaida, Hidenori Suzuki

**Affiliations:** 1 Department of Neurosurgery, Mie University Graduate School of Medicine, Tsu, Mie, Japan; 2 Department of Neurosurgery, Mie Chuo Medical Center, Tsu, Mie, Japan; Huazhong University of Science and Technology, CHINA

## Abstract

**Objective:**

This study aimed to predict recurrence after coil embolization of unruptured cerebral aneurysms with computational fluid dynamics (CFD) using porous media modeling (porous media CFD).

**Method:**

A total of 37 unruptured cerebral aneurysms treated with coiling were analyzed using follow-up angiograms, simulated CFD prior to coiling (control CFD), and porous media CFD. Coiled aneurysms were classified into stable or recurrence groups according to follow-up angiogram findings. Morphological parameters, coil packing density, and hemodynamic variables were evaluated for their correlations with aneurysmal recurrence. We also calculated residual flow volumes (RFVs), a novel hemodynamic parameter used to quantify the residual aneurysm volume after simulated coiling, which has a mean fluid domain > 1.0 cm/s.

**Result:**

Follow-up angiograms showed 24 aneurysms in the stable group and 13 in the recurrence group. Mann-Whitney U test demonstrated that maximum size, dome volume, neck width, neck area, and coil packing density were significantly different between the two groups (P < 0.05). Among the hemodynamic parameters, aneurysms in the recurrence group had significantly larger inflow and outflow areas in the control CFD and larger RFVs in the porous media CFD. Multivariate logistic regression analyses demonstrated that RFV was the only independently significant factor (odds ratio, 1.06; 95% confidence interval, 1.01–1.11; P = 0.016).

**Conclusion:**

The study findings suggest that RFV collected under porous media modeling predicts the recurrence of coiled aneurysms.

## Introduction

Coil embolization for cerebral aneurysms is widely used due to its minimally invasive nature and recent advancements in embolization devices. Surgical clipping reportedly features similar mortality rates as coiling but significantly higher periprocedural morbidity rates [1]. However, recanalization and re-treatment occur more often in coil embolization [2,3]. A larger aneurysmal dome or neck, minor recurrence noted on cerebral angiograms early after coil embolization, and lower coil packing density are significant risk factors for recanalization and re-treatment [4,5].

**Fig 1:**
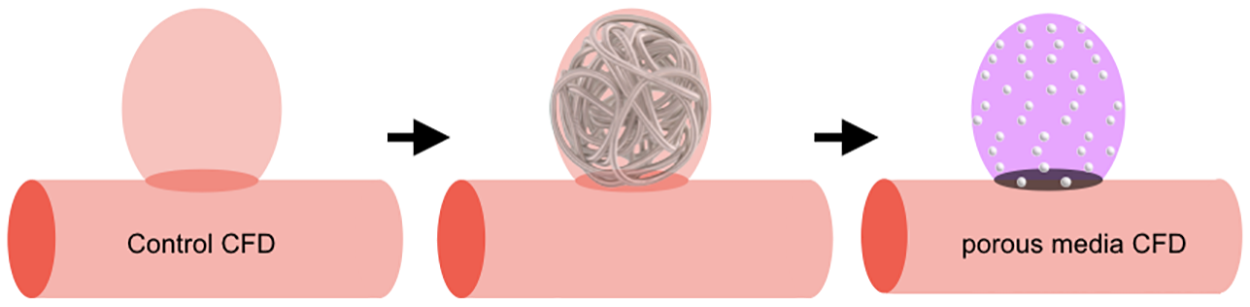
Schematic diagram of porous media modeling for coil embolization.

A recent study reported that high wall shear stress (WSS) and blood flow velocity were consistently observed on computational fluid dynamics (CFD) near the remnant neck of partially embolized aneurysms prone to future recanalization [6]. As CFD using a patient-specific model simulates the hemodynamics based on aneurysm geometry, the residual aneurysm and coil packing density would affect post-coiling hemodynamics. However, it has been difficult to simulate the hemodynamics of coiled aneurysms, because the accurate geometry of aneurysms after coiling could not be acquired using three-dimensional (3D) computed tomography (CT) angiography or digital subtraction angiography (DSA) due to metal artifacts. Instead, hemodynamics in coiled aneurysms have reportedly been simulated using numerical methods with porous media modeling [7]. Porous media has been used for modeling by determining particle porosity and by matching the size to the primary coil’s diameter (Fig 1). However, clinical usefulness of CFD with porous media modeling has never been examined. This method can simulate the hemodynamics after coiling. If a useful parameter is determined for predicting coiled aneurysm recurrence, this method may be used in pre-coiling planning. Our preliminary study using porous media modeling suggested that a novel parameter, residual flow volume (RFV), with a mean blood flow velocity > 1.0 cm/s in the aneurysm dome, could predict angiographic occlusion status after coiling [8]. This study aimed to evaluate the usefulness of RFV for predicting coiled aneurysm recurrence using a multivariate logistic regression analysis and the receiver operating characteristic (ROC) curve. This study would be the first step to prove a clinical usefulness of CFD with porous media modeling in pre-coiling planning.

## Materials and methods

The Institution Review Board of Mie University Graduate School of Medicine approved this retrospective study and waived the requirement for informed consent. Patient records and geometric data were anonymized prior to the analysis.

### Patient population and study protocol

We searched our database for all patients with cerebral aneurysms who underwent coil embolization between 2009 and 2013 at Mie University Hospital. Inclusion criteria were as follows: 1) saccular aneurysms treated with coil embolization without stent assist or parent artery occlusion; 2) follow-up DSA performed 6–12 months after coiling; and 3) accurate preoperative patient-specific geometry obtained from 3D rotational angiography or 3D CT angiography. A total of 36 patients with 37 unruptured aneurysms met the criteria and constituted this study population.

Medical charts and imaging studies were reviewed to record patient age and sex; aneurysm size and location; coil specification and packing density; and immediate and follow-up DSA findings. The placed coils included bare platinum, bioactive, and hydrogel-coated types. The total coil volume (CV) was calculated using the primary diameter of bare or bioactive coils (0.010–0.015 inches) or diameter after full expansion of hydrogel-coated coils (0.013–0.029 inches). Immediate and follow-up angiographic occlusion status was classified as complete occlusion (CO), residual neck (RN), or residual aneurysm (RA) based on Raymond’s classification. Classification of changes in angiographic occlusion status into stable or recurrence groups was based on comparisons of the occlusion status between immediately after coiling and 6–12 months later. An aneurysm was considered recurrent if completely occluded aneurysms on the initial DSA had partial recanalization in the neck and/or sac on the follow-up DSA. An aneurysm was also considered recurrent if a subtotally occluded aneurysm demonstrated an increased residual neck or residual aneurysm. Two independent observers blinded to the hemodynamic results evaluated and classified the aneurysms as described above.

### CFD analysis

The patient-specific geometries were generated as stereolithography (STL) from preoperative 3D rational angiography data that were obtained using an Allura imaging system (Philips Medical Systems, Best, Netherlands) or 3D CT angiography using an Aquilion One (Toshiba Medical Systems, Otawara, Japan). Vessels with diameters < 1 mm were excluded from the analysis (Magics 16.0; Matelialise Japan, Yokohama, Japan) because blood vessels with high Reynolds numbers had turbulent flow and were unsuitable for analysis under laminar flow. The STL was re-meshed to improve surface triangle quality (3-matic 6.0; Materialise Japan, Yokohama, Japan). The computational hybrid meshes were generated with tetrahedral and prism elements (ICEM CFD16.1; ANSYS, Inc., Canonsburg, PA, USA). Tetrahedral element sizes were 0.1–0.6 mm. Six prismatic boundary layers with a total thickness of 0.15 mm covered the vessel wall to locally ensure an accurate definition of the velocity gradient. Mesh dependency tests were performed in order to ensure the stability of simulation. A straight inlet extension was added to the inlet section to achieve a fully developed laminar flow. The surface geometry and fluid domain were divided into the aneurysm dome and the parent artery by the optimal neck plane corresponding to the line between the parent artery and ideally placed intra-aneurysmal coils. Maximum dome size, neck width, dome volume (DV), and neck area were measured by a CFX-Post (CFX CFD16.1; ANSYS, Inc.).

**Fig 2:**
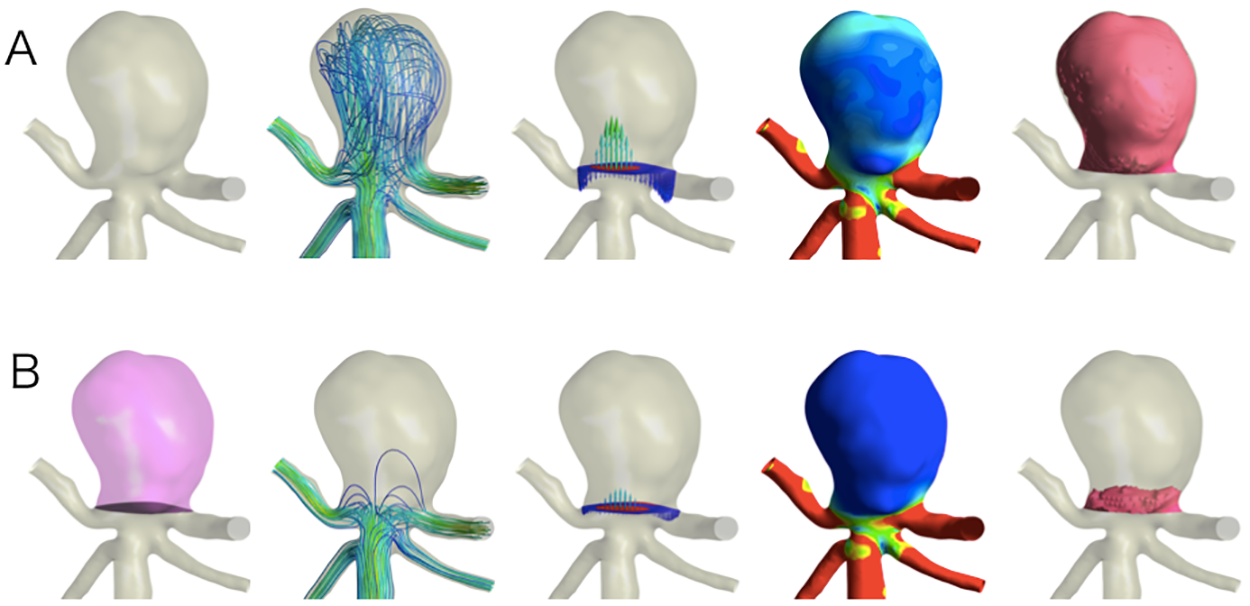
Representative cerebral aneurysm in the basilar artery (A-B). A, control computational fluid dynamics (CFD) model; B, porous media CFD. From left to right, segmentation of the neck plane (black) and intra-aneurysmal domain to define porous media (pink domain), streamline, inflow area (red), outflow area (blue), wall shear stress, and flow volume domain with a mean flow velocity > 1.0 cm/s (red domain) indicating residual flow volume (RFV) on the porous media CFD.

For the fluid domain, 3D incompressible laminar flow fields were obtained by solving the continuity and Navier-Stokes equations. Numeral modeling was performed using a commercially available CFD package (CFX CFD16.1; ANSYS, Inc.). Blood was assumed to be an incompressible Newtonian fluid with a blood density of 1056 kg/m^3^ and a blood dynamics viscosity of 0.0035 Pa·s. The typical flow waveform [9] of phase-contrast magnetic resonance imaging was scaled to achieve a physiological WSS. The steady flow analysis was assumed to be the mean flow volume rate for simplicity in pre-coiling (control CFD) (Fig 2A) and post-coiling (porous media CFD) (Fig 2B). A pulsatile flow analysis was performed of the porous media CFD. The time steps were 0.0001 seconds, and two pulsatile cycles were taken as output. Traction-free boundary conditions were applied to the outlets.

### Porous media model

In this study, the flow was categorized as that in the coil-free (as preoperative status) or coiled simulation. Flow in the control CFD was a model with Navier-Stroke equations and the equation of continuity given by

∇∙v=div∙ν=0

$$\frac{\partial \nu }{\partial t}+\left(\nu \cdot \nabla \right)\nu =-\frac{1}{\rho }\nabla p+\frac{\mu }{\rho }\nabla^{2}\nu +F$$

where ***v*** is the velocity of the flow, *p* is the pressure, *ρ* is the density, *μ* is the viscosity of the fluid, and *F* is the force.

The flow in the coiled regimes was simulated as a porous media modeling that obeys Darcy’s law. In these flow regimes, pressure is locally balanced with resistance forces such that

0=-∇p-Kν

where *K* is a constant of porous resistance and this is assumed to be a quasilinear function of the magnitude of velocity

K=α|v|+β

where *α* and *β* are coefficients for which the values are determined from Ergan’s equation [10,11], while the coefficient values of *α* and *β* are given as

$$\alpha =\frac{1.75\rho (1-\kappa )}{\kappa^{3}D_{P}}$$

$$\beta =\frac{150\mu (1-\kappa {)}^{2}}{\kappa^{3}D_{P}^{2}}$$

where *D*_*P*_ is the average particle diameter. Although various coils were included in this study (range of primary coil diameters: 0.010–0.029 inches), *D*_*P*_ was set to 0.010 inches (2.54 × 10^−4^ m) in all cases. *k* is the porosity determined as follows:

$$Coil\;packing\;density\;(\%)=\frac{CV}{DV}\times 100$$

$$\kappa =1-\frac{Coil\;packing\;density}{100}$$

WSS, normalized WSS, and flow velocity in fluid domain were calculated as mean aneurysm values. Inflow zone or outflow zone was identified based on vertical velocity vectors at the neck plane. In each zone, area and average velocity were calculated. RFV was calculated using porous media CFD. RFV was defined as the volume of fluid domain in the aneurysmal dome, which has a mean flow velocity > 1.0 cm/s (Fig 2B), according to our previous study [8]: among thresholds of 0.5, 1.0, 2.0, 5.0 and 10.0 cm/s tested, the threshold of 1.0 cm/s was the best predictor of 6–12-month post-coiling angiographic results of unruptured cerebral aneurysms.

### Statistical analysis

All values are expressed as mean (standard deviation). Statistical analyses were performed using SPSS Statics software (version 22.0; IBM, Chicago, IL, USA). We compared the variables between the stable and recurrence groups. Mann-Whitney U test was used to examine continuous variables, while Fisher’s exact test or the chi-square test was used to analyze categorical variables. The following variables were analyzed: patient age and sex; aneurysm location and subtype; coil specification; angiographic findings; morphological parameters; coil packing density; and hemodynamic parameters quantified by control CFD or porous media CFD. A univariate logistic regression analysis was performed to determine the association between post-coiling recurrence and significant parameters on the Mann-Whitney U tests. The cutoff value for the requirement for inclusion in the univariate logistic regression analysis was P < 0.05. A stepwise backward multivariate logistic regression analysis was then performed to determine the independent association of each factor with post-coiling recurrence. P values < 0.05 were considered statistically significant, while the area under the ROC curve (AUC) was used to determine the diagnostic accuracy of predicting recurrence post-coiling. A correlation between hemodynamics variables was verified using the Spearman rank correlation test. P values < 0.05 were considered to be significantly correlated.

## Results

### Patient characteristics and procedure specifications

**Fig 3:**
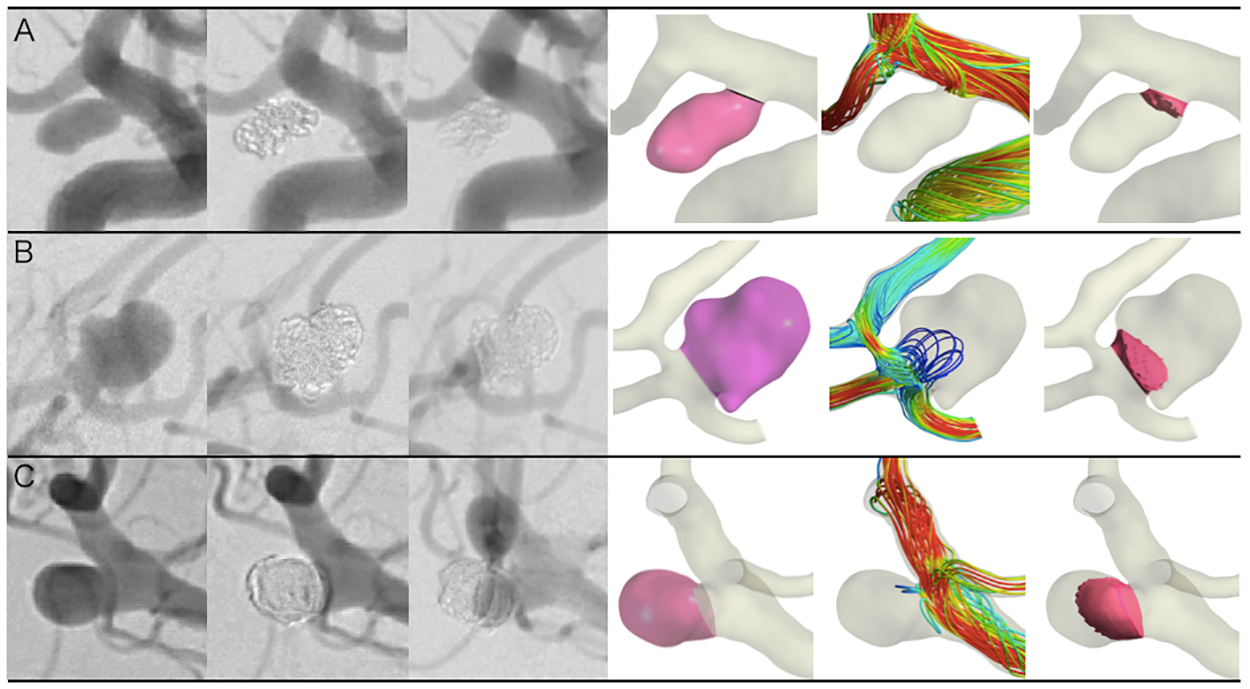
Representative cerebral aneurysms. A, right internal carotid artery aneurysm with complete occlusion; B, anterior communicating artery aneurysm with residual neck; C, right middle cerebral artery aneurysm with residual aneurysm on follow-up angiogram. From left to right, pre-coiling angiograms, post-coiling immediate angiograms, follow-up angiograms, segmentation of porous medium (pink domain), streamline at the post-coil embolization under the porous media model, and residual flow volume (RFV) with a mean flow velocity > 1.0 cm/s (red domain). Aneurysm volume (A:B:C = 54.8:255.4:146.7 mm^3^), coil packing density (38.7:24.3:24.7%), and RFV (3.7:27.1:56.5 mm^3^), respectively.

The cohort included 25 (67.6%) women and 12 (32.4%) men with a mean age of 62.8 (9.1) years. Twenty-four aneurysms were in the internal carotid artery, seven in the posterior circulation, five in the anterior communicating artery, and one in the middle cerebral artery. The mean aneurysm size was 7.7 (4.0) mm. Coiling procedures were performed with bare-platinum coils only (n = 19), a combination of bare and bioactive coils (n = 12), or bare and hydrogel-coated coils (n = 6). Mean coil packing density was 29.7 (10.8) %. On the immediate angiograms, 14 aneurysms were classified as CO, 14 as RN, and nine as RA. On follow-up DSA images, 19 aneurysms were distinguished as CO, 11 as RN, and seven as RA. As a result, 24 (64.9%) aneurysms were classified into the stable group, while 13 (35.1%) were classified into the recurrence group. In the stable group, the occlusion status of 14 (37.8%) aneurysms improved (seven from RN to CO, four from RA to CO, and three from RA to RN) but remained unchanged in 10 (27.0%; CO in eight, and RN in two). In the recurrence group, the occlusion status deteriorated from CO to RN in four, from CO to RA in two, and from RN to RA in three, while an increasing residual neck and residual aneurysm were observed in two, respectively. Four aneurysms were re-treated in the recurrence group. Control CFD and porous media CFD analyses were performed for all aneurysms (Fig 3).

### Nonparametric statistics between the stable and recurrence groups

All parameters were compared between the stable and recurrence groups. The recurrence group had significantly larger maximum size (6.8 versus 8.7 mm; P = 0.018), dome volume (126.1 versus 431.6 mm^3^; P = 0.003), neck width (4.1 versus 6.0 mm; P = 0.006), neck area (12.0 versus 32.9 mm^2^; P = 0.005), and lower coil packing density (33.1 versus 23.4%; P = 0.001) (Table 1). There were no significant differences in age, sex, aneurysm subtype, coil specification, immediate angiographic results, or follow-up angiogram term. Among the hemodynamic variables with control CFD, the recurrence group had significantly larger inflow (4.4 versus 13.2 mm^2^; P = 0.002) and outflow (7.5 versus 19.7 mm^2^; P = 0.005) areas (Table 2). On the other hand, porous media CFD revealed that the recurrence group had significantly larger RFV (13.9 versus 97.2 mm^3^; P < 0.001). There were no significant differences in WSS, normalized WSS, or flow velocity.

**Table 1:** Characteristics of unruptured cerebral aneurysms treated by coil embolization.

	Stable group (n = 24)	Recurrence group (n = 13)	P value
**Age (years)**	61.5 ± 8.1 (38–73)	65.2 ± 10.8 (45–85)	ns*
**Sex (M/F)**	9/15	3/10	ns^†^
**Aneurysm size**			
Maximum size (mm)	6.8 ± 3.3 (4.1–18.8)	8.7 ± 3.0 (4.7–13.5)	0.018*
Dome volume (mm^3^)	126.1 ± 241.3 (8.9–1191.0)	431.6 ± 668.2 (35.8–2511.0)	0.003*
**Neck orifice size**			
Neck width (mm)	4.1 ± 1.5 (2.4–7.2)	6.0 ± 2.2 (3.2–11.2)	0.006*
Neck area (mm^2^)	12.0 ± 8.7 (3.9–35.3)	32.9 ± 39.5 (6.8–153.0)	0.005*
**Aneurysm subtype**			ns^†^
Sidewall type	17 (71)	5 (38)	
Terminal type	7 (29)	8 (62)	
**Coil specification**			ns^†^
Bare platinum coil only	9 (38)	10 (78)	
Bio-active coil	10 (42)	2 (15)	
Hydrogel-coated coil	5 (21)	1 (8)	
**Coil packing density (%)**	33.1 ± 11.0 (17.3–62.7)	23.4 ± 7.1 (13.3–38.8)	0.001*
**Immediate angiogram result**			ns^†^
Complete occlusion	8 (33)	6 (46)	
Residual neck	9 (38)	5 (38)	
Residual aneurysm	7 (29)	2 (15)	
**Follow-up angiogram result**			<0.001^†^
Complete occlusion	19 (79)	0	
Residual neck	5 (21)	6 (46)	
Residual aneurysm	0	7 (54)	
**Follow-up angiogram term (months)**	8.7 ± 3.2 (6–12)	8.4 ± 3.0 (6–12)	ns*

M/F, male to female ratio; ns, not significant.

Data are expressed as mean ± standard deviation (range) or number of cases (% of cases).

*Estimated using Mann-Whitney U tests.

^†^Estimated using Fisher exact tests or chi-square tests as appropriate.

**Table 2:** Comparison of hemodynamic parameters using computational fluid dynamics analyses.

Parameter	Stable group (n = 24)	Recurrence group (n = 13)	P value*
**Control CFD**			
** WSS (Pa)**	2.96 ± 3.04 (0.14–12.51)	3.41 ± 2.72 (0.35–9.68)	ns
** Normalized WSS**	0.33 ± 0.22 (0.02–0.80)	0.35 ± 0.14 (0.11–0.58)	ns
** Flow velocity (cm/s)**	7.06 ± 5.32 (0.58–21.59)	9.58 ± 7.07 (2.31–29.34)	ns
** Inflow area (mm**^**2**^**)**	4.42 ± 3.02 (1.23–11.09)	13.21 ± 17.65 (2.44–69.52)	0.002
** Outflow area (mm**^**2**^**)**	7.54 ± 5.89 (2.37–24.26)	19.72 ± 22.36 (4.33–83.46)	0.005
** Inflow velocity (cm/s)**	27.59 ± 14.63 (5.36–61.08)	30.05 ± 16.18(7.72–61.47)	ns
** Outflow velocity (cm/s)**	16.67 ± 9.24 (2.00–36.46)	18.17 ± 10.86 (5.46–45.10)	ns
**Porous media CFD**			
** Wall shear stress (Pa)**	0.45 ± 0.66 (0.03–3.19)	0.52 ± 0.46 (0.06–1.55)	ns
** Normalized WSS**	0.05 ± 0.04 (0.003–0.18)	0.05 ± 0.03 (0.02–0.12)	ns
** Flow velocity (cm/s)**	0.88 ± 1.13 (0.04–5.53)	1.25 ± 1.00 (0.22–3.01)	ns
** Inflow area (mm**^**2**^**)**	5.08 ± 3.35 (0.81–10.68)	9.33 ± 5.26 (2.86–19.91)	ns
** Outflow area (mm**^**2**^**)**	6.90 ± 5.56 (2.39–24.73)	23.60 ± 36.44 (3.80–139.8)	ns
** Inflow velocity (cm/s)**	16.59 ± 15.49 (0.65–55.94)	23.02 ± 13.60 (7.00–49.27)	ns
** Outflow velocity (cm/s)**	11.03 ± 11.50 (0.60–43.91)	11.64 ± 7.73 (1.88–31.49)	ns
** RFV (mm**^**3**^**)**	13.91 ± 14.20 (0.65–61.43)	97.19 ± 117.48 (7.95–322.5)	<0.001

CFD, computational fluid dynamics; WSS, average wall shear stress at the aneurysmal wall; flow velocity, mean flow velocity in the aneurysmal dome; RFV, intra-aneurysmal residual flow volume with an average flow velocity > 1.0 cm/s; ns, not significant.

Data are expressed as mean ± standard deviation (range).

*Estimated using Mann-Whitney U tests.

### Univariate and multivariate logistic regression analyses to predict post-coiling recurrence

In the univariate logistic regression analyses, larger neck width, neck area, inflow area, RFV, and lower coil packing density were significant factors for the recurrence. However, maximum size, dome volume, and outflow area were not significant factors (Table 3). Before the multivariate analyses were performed, intercorrelations were examined between significant parameters in the Mann-Whitney U tests using a Spearman rank correlation test: maximum size and dome volume (correlation coefficient: R = 0.946, P < 0.001), neck width and neck area (R = 0.975, P < 0.001), inflow and outflow areas in control CFD (R = 0.930, P < 0.001), and neck and inflow areas (R = 0.972, P < 0.001) were significantly correlated. RFV was not correlated with dome volume (R = 0.590, P > 0.05). Thus, in addition to coil packing density and RFV, dome volume and inflow area were considered independent variables because they had the lowest P values among the significantly correlated parameters. On the multivariate logistic regression analyses, only RFV was a significant predictor of recurrence (odds ratio [OR], 1.060; 95% confidence interval [CI], 1.011–1.112; P = 0.016) (Table 3). On the analyses of the ROC curve for RFV, the AUC was 0.86 (95% CI, 0.74–0.98) and the cut-off value was 20.4 mm^3^, with a sensitivity of 0.77 and a specificity of 0.79. RFV had the highest AUC value of the parameters (Table 4).

**Table 3:** Univariate and multivariate logistic regression analyses of independent parameters with respect to recurrence after coiling.

Parameter	Mann-Whitney U test	Univariate logistic regression		Multivariate logistic regression	
	P	P	OR (95% CI)	P	OR (95% CI)
**Morphological**					
Maximum size (mm)	0.018	0.093	1.22 (0.97–1.54)		
Dome volume (mm^3^)	0.003	0.137	1.00 (0.99–1.01)	0.442	1.00 (0.99–1.01)
Neck width (mm)	0.006	0.014	1.87 (1.13–3.08)		
Neck area (mm^2^)	0.005	0.038	1.09 (1.01–1.17)		
**Coil**					
Coil packing density (%)	0.001	0.016	0.85 (0.75–0.97)	0.367	0.94 (0.82–1.08)
**Control CFD**					
Inflow area (mm^2^)	0.002	0.021	1.30 (1.04–1.62)	0.599	1.10 (0.77–1.59)
Outflow area (mm^2^)	0.005	0.059	1.11 (0.99–1.25)		
**Porous media CFD**					
RFV (mm^3^)	0.001	0.016	1.06 (1.01–1.11)	0.016	1.06 (1.01–1.11)

OR, odds ratio; CI, confidence interval; CFD, computational fluid dynamics; RFV, intra-aneurysmal residual flow volume with a mean flow > 1.0 cm/s.

**Table 4:** Receiver operating characteristic (ROC) curve analyses of coiled aneurysm recurrence.

	AUC	95% CI	Sensitivity	Specificity	Cut-off value
**Maximum size**	0.74	0.57–0.90	0.69	0.71	6.8 mm
**Dome volume**	0.80	0.65–0.94	0.77	0.71	79.5 mm^3^
**Neck width**	0.77	0.62–0.93	0.85	0.75	4.3 mm
**Neck area**	0.83	0.70–0.96	0.84	0.79	13.1 mm^2^
**Coil packing density**	0.81	0.65–0.97	0.77	0.71	27.4%
**Inflow area**	0.77	0.61–0.92	0.77	0.75	5.3 mm^2^
**Outflow area**	0.79	0.63–0.94	0.85	0.75	7.9 mm^2^
**RFV**	0.86	0.74–0.98	0.77	0.79	20.4 mm^3^

AUC, area under the ROC curve; CI, confidence interval; RFV, intra-aneurysmal residual flow volume with a mean flow velocity > 1.0 cm/s.

### RFV by transient analysis

Porous media CFD was also performed using transient analysis. The RFV values by transient analysis (RFV_trans_) were calculated as mean velocity in one cardiac cycle. The RFV_trans_ values were significantly lower than the RFV (22.1 versus 43.1 mm^3^, P < 0.001). Similar to the RFV, however, the recurrence group had significantly larger RFV_trans_ values than the stable group (48.6 versus 7.7 mm^3^, P < 0.001) (Table 5). In addition, multivariate logistic regression analyses showed that RFV_trans_ was a single significant predictor of recurrence (OR, 1.110; 95% CI, 1.015–1.213; P = 0.022). Spearman rank-correlation coefficient demonstrated that RFV_trans_ was well correlated with RFV (R = 0.982, P < 0.0001). On the contrary, the mean solution time of the transient analysis was about 250 times longer than that of the steady-state analysis (25 hours, 34 minutes versus 7.1 minutes).

**Table 5:** Comparison of hemodynamic parameters using computational fluid dynamics analyses on transient analysis.

Parameter	Stable group (n = 24)	Recurrence group (n = 13)	P value*
**Porous media CFD by transient analysis**			
WSS (Pa)	0.47 ± 0.69 (0.04–3.32)	0.55 ± 0.49 (0.06–1.70)	ns
Normalized WSS	0.05 ± 0.04 (0.01–0.18)	0.05 ± 0.03 (0.02–0.12)	ns
Flow velocity (cm/s)	0.89 ± 1.37 (0.02–6.68)	1.25 ± 1.14 (0.16–3.92)	ns
Inflow area (mm^2^)	5.18 ± 3.27 (0.82–11.2)	9.32 ± 5.33 (2.97–20.6)	ns
Outflow area (mm^2^)	6.79 ± 5.67 (1.03–24.7)	23.62 ± 36.8 (3.75–140.0)	ns
Inflow velocity (cm/s)	10.34 ± 10.6 (0.33–38.1)	13.90 ± 8.76(3.62–32.9)	ns
Outflow velocity (cm/s)	7.15 ± 7.66 (0.28–28.7)	7.26 ± 4.89 (1.15–18.4)	ns
RFV_trans_ (mm^3^)	7.70 ± 7.54 (0.58–27.9)	48.58 ± 58.7 (3.31–185.7)	<0.001

CFD, computational fluid dynamics; WSS, average wall shear stress at aneurysmal wall; flow velocity, mean flow velocity in the aneurysmal dome; RFV_trans_, intra-aneurysmal residual flow volume with a mean flow velocity > 1.0 cm/s; ns, not significant.

Data are expressed as mean ± standard deviation (range).

*Estimated using Mann-Whitney U tests.

## Discussion

This study was the first to demonstrate that RFV based on porous media CFD using preoperative aneurysm geometry could predict aneurysm recurrence after coil embolization. This study also confirmed that recurrence is prone to occur in aneurysms with a larger dome or neck size, or a lower coil packing density [12–14]. However, multivariate and ROC curve analyses showed that RFV is the most important and significant predictor of recurrence among these morphological parameters and coil packing density in this study.

Endovascular coiling has been an important treatment modality for unruptured cerebral aneurysms for its less invasive nature. However, the drawback of endovascular coiling includes a greater risk of recurrence and retreatment compared to surgical clipping [15,16]. To prevent post-coiling recurrence, hydrogel-coated and flexible large coils are used to achieve a high packing density, especially in cases of aneurysms with high recurrence risk. However, reported optimal coil packing density cut-off values are variable: in one study, it was 25% (sensitivity, 68.8%; specificity, 53.3%) [17]; in another study, appropriate values were 20–36% [18,19]. Moreover, an in vitro study showed that aneurysm geometry affected post-coiling intra-aneurysmal hemodynamics despite similar coil packing density [20]. To resolve the issue of post-coiling recurrence, it may be required to determine the optimal packing density per aneurysm rather than a predetermined uniform one.

Recent studies using image-based CFD modeling have revealed potential connections between the hemodynamic properties and aneurysm initiation, growth, and rupture. Leo et al [6] reported that partially embolized aneurysms with a high WSS and high blood flow velocity at the remnant neck likely recurred during follow-up. These results suggested that an insufficient flow reduction would result in post-coiling recurrence. Hemodynamics after coiling may play a key role in aneurysmal recurrence as well. However, it is difficult to acquire the accurate residual aneurysm geometry because the placed coils are associated with serious metal artifacts.

**Fig 4:**
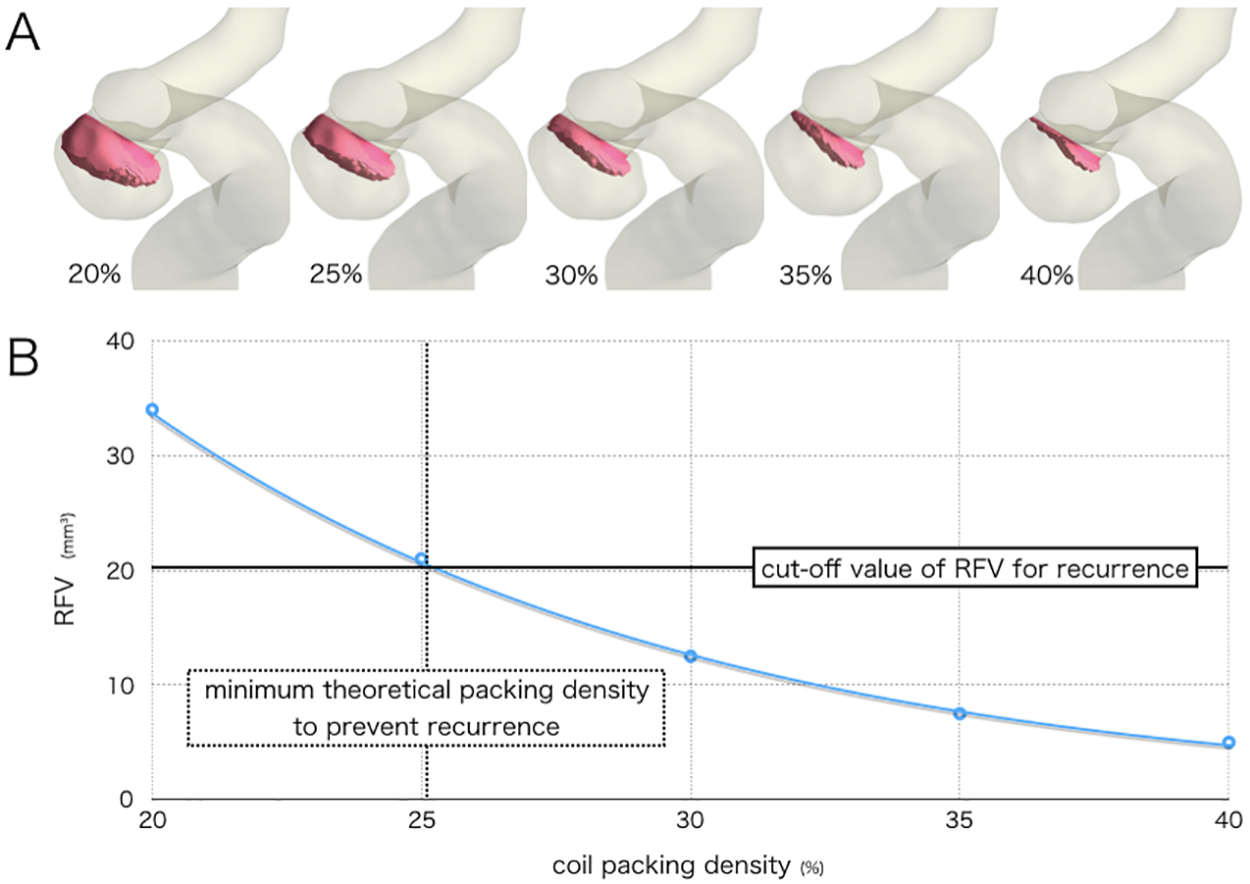
Preoperative simulation of a right internal carotid artery aneurysm. A, flow volume domain with a mean flow velocity > 1.0 cm/s (red domain) indicating residual flow volume (RFV) simulated by various coil packing densities. B, graph showing that the coil packing density must be >25% by RFV values in the preoperative simulation to prevent aneurysm recurrence.

CFD using porous media modeling has been used to simulate the hemodynamics after aneurysm coiling [21,22]. In this method, the flow in the coiled regimes is modeled as a porous media. Porous media modeling consists of uniformly placed particles, the sizes of which match the primary coil diameter and porosity corresponds with coil packing density. To our knowledge, the clinical usefulness of CFD porous media modeling has not been examined. Here, we performed CFD using porous media modeling to clarify the correlation between hemodynamics and post-coiling recurrence. A novel hemodynamic parameter, RFV, indicates intra-aneurysmal volume of the fluid domain with a mean flow velocity > 1.0 cm/s. The threshold was determined in our previous study, in which the threshold flow velocity of 0.5–10.0 cm/s was tested to predict post-coiling angiographic aneurysm occlusion status; in this test, 1.0 cm/s was determined to be optimal [8]. This study’s findings clearly suggested that RFV is a useful parameter for predicting coiled aneurysm recurrence, and that post-coiling aneurysm recurrence may depend on the volume of post-coiling intra-aneurysmal residual blood flow with a velocity greater than a certain threshold, not on pre-coiling blood flow velocity within the aneurysm dome that varies greatly depending on aneurysms. RFV may also be used in pre-coiling planning; that is, the coil packing density can be calculated preoperatively for RFV to keep it at <20.4 mm^3^, the RFV cut-off value used to prevent recurrence that was determined using the ROC curve analyses (Table 4, Fig 4). Thus, the optimal coiling packing density per aneurysm can be determined preoperatively. This study also showed that a steady-state analysis is sufficient for calculating RFV and requires only about 7 minutes. This is a merit of using RFV in the clinical setting.

This study has several limitations. First, the porous media modeling did not reflect the primary diameters and distribution of actually placed coils. The coil packing density of each aneurysm was set as porosity per aneurysm, but the particle size was set uniform, i.e., 2.54 × 10^−4^ m (0.010 inches) in all aneurysms. The discrepancy of coil distribution between actually placed coils in each aneurysm and uniform density in porous media modeling might bias our results. Second, for the calculation of RFV, it is necessary to set the threshold velocity, but the optimal RFV threshold may change by changing numerical modeling such as blood flow at the inlet. In addition, we assumed vessel walls to be rigid, blood was modeled as a Newtonian fluid and boundary conditions were taken from a typical wave. Newton assumption may underestimate the WSS and flow velocity in the low shear rate field. Therefore, there is a theoretical possibility that the differences could bias our results. Thus, better numerical simulation for a proper modeling of CFD using porous media should be determined in the future experiments. Third, RFV could not be applied to aneurysms with dome volume below the RFV cut-off value, although such small aneurysms hardly recur after coiling. Although we consider that the absolute value of RFV is suitable for predicting coiled aneurysm recurrence at this time, it needs to investigate whether some kind of normalized RFV depending on a broad range of pre-coiling aneurysm dome volume is more useful in the future. Finally, this retrospective study included a small number of cases; thus, further large-scale prospective studies are needed to confirm our findings.

## Conclusion

This study showed that RFV may be a more useful parameter for predicting post-coiling recurrence than previously reported risk factors. RFV can also be used in pre-coiling planning, contributing to more effective coil embolization of cerebral aneurysms.

## Supporting information

S1 File Tables 1 and 2 information file. (XLSX)

## References

[1] McDonald JS, McDonald RJ, Fan J, Kallmes DF, Lanzino G, Cloft HJ; Comparative effectiveness of unruptured cerebral aneurysm therapies propensity score analysis of clipping versus coiling. *Stroke*. 2013; 44: 988–994. doi: 10.1161/STROKEAHA.111.000196 23449260 10.1161/STROKEAHA.111.000196

[2] Campi A, Ramzi N, Molyneux AJ, Summers PE, Kerr RS, Sneade M, et al; Retreatment of ruptured cerebral aneurysms in patients randomized by coiling or clipping in the international subarachnoid aneurysm trial (ISAT). *Stroke*. 2007; 38: 1538–1544. doi: 10.1161/STROKEAHA.106.466987 17395870 10.1161/STROKEAHA.106.466987

[3] Lad SP, Babu R, Rhee MS, Franklin RL, Ugiliweneza B, Hodes J, et al; Long-term economic impact of coiling vs clipping for unruptured intracranial aneurysms. *Neurosurgery*. 2013; 72: 1000–1013. doi: 10.1227/01.neu.0000429284.91142.56 23612602 10.1227/01.neu.0000429284.91142.56

[4] Chalouhi N, Bovenzi CD, Thakkar V, Dressler J, Jabbour P, Starke RM, et al; Long-term catheter angiography after aneurysm coil therapy: results of 209 patients and predictors of delayed recurrence and retreatment. *J Neurosurg*. 2014; 5: 1–5. 10.3171/2014.7.JNS132433 25192480

[5] Sluzewski M, van Rooij WJ, Slob MJ, Bescós JO, Slump CH, Wijnalda D; Relation between aneurysm volume, packing density, and compaction in 145 cerebral aneurysms treated with coil. *Radiology*. 2005; 231: 653–658. 10.1148/radiol.2313030460 15118115

[6] Luo B, Yang X, Wang S, Li H, Chen J, Yu H, et al; High shear stress and flow velocity in partially occlusion aneurysms prone to recanalization. *Stroke*. 2011; 42: 745–753. doi: 10.1161/STROKEAHA.110.593517 21233477 10.1161/STROKEAHA.110.593517

[7] Cha KS, Balaras E, Lieber BB, Sadasivan C, Wakhloo AK; Modeling the interaction of coils with the local blood flow after coil embolization of intracranial aneurysms. *J Biomech Eng*. 2007; 129: 873–879. doi: 10.1115/1.2800773 18067391 10.1115/1.2800773

[8] Umeda Y, Ishida F, Tsuji M, Furukawa K, Sano T, Toma N, et al; Computational fluid dynamics (CFD) analysis using porous media modeling predicts angiographic occlusion status after coiling of unruptured cerebral aneurysms–preliminary study. *JNET*. 2015; 9: 69–77.

[9] Ford MD, Alperin N, Lee SH, Holdsworth DW, Steinman DA; Characterization of volumetric flow rate waveforms in the normal internal carotid and vertebral arteries. *Physiol Meas*. 2005; 26: 477–488. doi: 10.1088/0967-3334/26/4/013 15886442 10.1088/0967-3334/26/4/013

[10] Ergun S; Fluid Flow through packed columns. *Chem Eng Prog*. 1952; 48: 85–94.

[11] Akgiray Ö, Saatçı AM; A new look at filter backwash hydraulics. *Water Sci Technol*. 2001; 1, 65–72.

[12] Soeda A, Sakai N, Sakai H, Iihara K, Nagata I; Endovascular treatment of asymptomatic cerebral aneurysms: anatomic and technical factors related to ischemic events and coil stabilization. *Neuro Med Chir (Tokyo)*. 2004; 44: 456–465. 10.2176/nmc.44.456 15600280

[13] Tan IYL, Agid RF, Willinsky RA; Recanalization rates after endovascular coil embolization in a cohort of matched ruptured an unruptured cerebral aneurysms. *Interv Neuroradiol*. 2011; 17: 27–35. doi: 10.1177/159101991101700106 21561556 10.1177/159101991101700106 PMC3278032

[14] Kawanabe Y, Sadato A, Taki W, Hashimoto N; Endovascular occlusion of intracranial aneurysms with Guglielmi detachable coils: correlation between coil packing density and coil compaction. *Acta Neurochir (Wien)*. 2001; 143: 451–455. 11482694 10.1007/s007010170073

[15] Kwon SC, Kwon OK; Endovascular coil embolization of unruptured intracranial aneurysms: a Korean multicenter study. *Acta Neurochir*. 2014; 156: 847–854. doi: 10.1007/s00701-014-2033-9 24610449 10.1007/s00701-014-2033-9

[16] Schaafsma JD, Sprengers ME, van Rooij WJ, Sluzewski M, Majoie CB, Wermer MJ, et al; Long-term recurrent subarachnoid hemorrhage after adequate coiling versus clipping of ruptured intracranial aneurysms. *Stroke*. 2009; 40: 1758–1763. doi: 10.1161/STROKEAHA.108.524751 19286603 10.1161/STROKEAHA.108.524751

[17] Ikeda G, Sonobe M, Kato N, Yamazaki T, Kasuya H, Nakai Y, et al; Correlation between rupture or retreatment and the volume embolization ration after coil embolization. *Surg Cereb Stroke*. 2013; 41: 440–446.

[18] Shimano H, Nagasawa S, Miyatake SI, Kawanishi M, Yamaguchi K, Kawabata S, et al; Model analysis of coil embolization of cerebral aneurysms: prediction of thrombus formation in aneurysms based in the coil embolization rate. *Neuro Res*. 2006; 28: 172–176. 10.1179/016164106X98044 16551435

[19] Yagi K, Satoh K, Satomi J, Matsubara S, Nnagahiro S; Evaluation of aneurysm stability after coil embolization with Guglielmi detachable coils: correlation between long term stability and volume embolization ratio. *Neuro Med Chir (Tokyo)*. 2005; 45: 561–565. 10.2176/nmc.45.561 16308514

[20] Babiker MH, Gonzalez LF, Albuquerque F, Collins D, Elvikis A, Frakes DH; Quantitative effects of coil packing density on cerebral aneurysm fluid dynamics: an in vitro steady flow study. *Ann Biomed Eng*. 2010; 38: 2293–2301. doi: 10.1007/s10439-010-9995-4 20306135 10.1007/s10439-010-9995-4

[21] Mitsos AP, Kakalis NM, Ventikos YP, Byrne JV; Hemodynamic simulation of aneurysm coiling in an anatomically accurate computational fluid dynamics model: technical note. *Neuroradiology*. 2008; 50: 341–347. doi: 10.1007/s00234-007-0334-x 18043912 10.1007/s00234-007-0334-x

[22] Otani T, Nakamura M, Fujinaka T, Hirata M, Kuroda J, Shibano K, et al; Computational fluid dynamics of blood flow in coil-embolized aneurysms: effect of packing density on flow stagnation in an idealized geometry. *Med Biol Eng Comput*. 2013; 51: 901–910. doi: 10.1007/s11517-013-1062-5 23529587 10.1007/s11517-013-1062-5

